# Role of Relative Humidity in Processing and Storage of Seeds and Assessment of Variability in Storage Behaviour in *Brassica* spp. and *Eruca sativa*

**DOI:** 10.1155/2013/504141

**Published:** 2013-12-30

**Authors:** A. Suma, Kalyani Sreenivasan, A. K. Singh, J. Radhamani

**Affiliations:** ^1^National Bureau of Plant Genetic Resources, Regional Station, Thrissur, Kerala 680656, India; ^2^National Bureau of Plant Genetic Resources, Pusa Campus, New Delhi 110012, India; ^3^Germplasm Conservation Division, National Bureau of Plant Genetic Resources, Pusa Campus, New Delhi 110012, India

## Abstract

The role of relative humidity (RH) while processing and storing seeds of *Brassica* spp. and *Eruca sativa* was investigated by creating different levels of relative humidity, namely, 75%, 50%, 32%, and 11% using different saturated salt solutions and 1% RH using concentrated sulphuric acid. The variability in seed storage behaviour of different species of *Brassica* was also evaluated. The samples were stored at 40 ± 2°C in sealed containers and various physiological parameters were assessed at different intervals up to three months. The seed viability and seedling vigour parameters were considerably reduced in all accessions at high relative humidity irrespective of the species. Storage at intermediate relative humidities caused minimal decline in viability. All the accessions performed better at relative humidity level of 32% maintaining seed moisture content of 3%. On analyzing the variability in storage behaviour, *B. rapa* and *B. juncea* were better performers than *B. napus* and *Eruca sativa*.

## 1. Introduction

The *Brassica* species have been cultivated in India since Vedic times. They are being used for many purposes, that is, edible oil, vegetable, fodder preparation and seasoning of food articles, and so forth. They have also been used as medicine and condiments. The small round seeds of *Brassicas* contain on average 37% of oil and more than 40% of protein in the seed meal, though it is mainly used as an oilseed crop. The *Brassica* species complex is generally referred to as rapeseed mustard, which is the second most important edible oilseed crop of India after groundnut. The cultivation of rapeseed mustard, which was earlier confined to the northern belt, has now spread to nontraditional areas in western and southern regions of the country. *Eruca sativa*, a member of Brassicaceae family, is an underutilized oilseed crop.

Availability of genetic diversity in the gene pool of cultivated species is key for a planned genetic improvement and variety development programme. In *Brassica*, availability of genetic diversity has been one of the major constraints. This is because the sizable collection available in the country does not represent the total spectrum of variability and because of the fact that, despite recognition to the importance of genetic diversity, it is being eroded because of poor conservation efforts. In case of cultivated plant species, where most of the genetic diversity is confined to the farmer's fields, *ex situ* conservation of plant genetic resources is the most common method for conservation. Appropriate conditions have to be identified to restrict the deterioration of seed during processing and to prolong the life of the seed in storage. Therefore, considerable interest has been shown to investigate most suitable seed characteristics and storage conditions for prolonged germplasm conservation [1–3], as long term storage may adversely affect the regeneration capabilities of the seed. The moisture content of the harvested seed is influenced by the surrounding relative humidity and the temperature conditions, the two major factors that control the longevity of the seed in processing and storage. Therefore, the seed processing and storage problems are common in tropical countries like India, which has predominantly hot and humid tropical and subtropical conditions with great variation in relative humidities and temperatures throughout the year (as per the season: summer, winter, and rainy), including biennial variation. Frequent fluctuation and relative humidity make the processing and storage of seeds difficult to minimize the loss of viability and change in genetic integrity of the seed and thereby conservation of genetic resources.

Research on the seed storage has corroborated that high temperature and relative humidity conditions accelerate the deterioration of the seed and thereby cause ageing. Therefore, exposure of seed to high temperature and high moisture conditions will cause ageing and has been commonly used for accelerated ageing of seeds in the laboratory. Such treatments to seeds prior to germination may yield useful information concerning the loss of seed viability, vigour, and longevity of viability [4] and may be used as a tool for predicting the relative seed-longevity. Thus deterioration would occur relatively slowly at low moisture and temperature. Hence, seed drying in case of orthodox seeds is an important step in seed processing. The seeds absorb or lose moisture till the vapor pressure of seed moisture and atmospheric moisture reach equilibrium [5]. However, experiments have shown that there are limits to the beneficial effects of drying and that drying below a critical level of moisture content will not improve longevity [6] and may even have detrimental effect on seed survival during processing and storage [7].

The type of storage component in the seed also influences the equilibration of seed moisture content. Nutrients stored in the seeds are mainly sugar, starch, protein, and fat (oil). The four components differ in their water affinity, sugar being the most hygroscopic (binding most water) followed by protein, starch, and oil. For fully hygroscopic seeds, oil content is a major factor that determines the attainment of final equilibrium seed moisture content. The role of oil is passive, acting as a constant, but it is hydrophobic element of the seed weight; consequently seeds with high oil contents equilibrate to lower moisture contents faster under the same conditions than seeds with lower oil contents. Because of the differences in anatomical structure and storage components of seeds, the equilibrium moisture content differs between species. Recognizing the value of critical moisture level, an insufficient drying may result in less than maximum longevity potential, while overdrying may result in waste of energy without any positive effect. Therefore, there is a need to investigate the effect of temperature and relative humidity for equilibration of seed moisture content to desirable levels. In addition species as *Brassica* are rich in oil contents, it could also be understood how the composition of oil content in the seed effects the drying of seed during processing and seed-viability during storage. In the present investigation an effort was made to investigate the role of relative humidity while processing and storaging *Brassica* seeds and to assess and analyze the variability in seed storage capacity of various species of *Brassica* and *Eruca sativa* under different relative humidities.

## 2. Material and Methods

The seed materials used in the present study include three accessions each of *Brassica* spp., namely, *B. juncea* (IC355331, IC241634, and IC241632), *B. napus *(IC241650, IC355318, and IC355321), and *B. rapa* (IC342764, IC241661, and IC355302), and two accessions of *Eruca sativa* (RTM-314 and TMLC-3) (a related genus of *Brassica*). The seeds were collected and procured from National Bureau of Plant Genetic Resources, New Delhi, India.

In order to investigate the role of ambient relative humidity during seed drying and transit storage, seeds were stored over saturated solutions of various salts for maintaining different levels of relative humidity. These were sodium chloride (75%), calcium nitrate (50%), magnesium chloride (32%), and lithium chloride (11%). The lowest relative humidity of 1% was created using concentrated sulphuric acid. The relative humidities were established based on the method of Winston and Bates [8] using specific standard salt solutions while the temperature was maintained using incubators set at the required temperature and relative humidities were monitored with the help of hygrothermograph. The storage conditions were kept constant and the variation in temperature and RH did not exceed 3–5%, respectively. The samples were stored in the respective humidity in sealed containers, which were then transferred to an incubator, maintained at 40 ± 2°C. The seeds were removed at an interval of one month for LiCl, MgCl_2_, and Ca(NO_3_)_2_, ten days for H_2_SO_4_, and fourteen days for NaCl up to three months. Various physiological parameters were estimated to assess the effect of relative humidity on seed drying and transit storage. Immediately before germination test all seeds except those maintained at 50 and 75% RH were humidified above water at 20°C for 24 hours in order to avoid imbibitional damage. Germination tests were conducted using 25 seeds in three replications. Seeds were plated in petri plates with wet filter paper and kept in germinator maintaining adequate humidity and temperature of 20 ± 2°C [9]. Germination count was taken every day up to the seventh day. Seeds were considered to be germinated if 1 mm radicle had emerged. Germinated seeds were removed every day. Germination percentage was recorded on the basis of normal seedlings. Speed of germination was estimated along with standard germination test. The humidity and temperature of germinator where samples were kept for speed of germination were the same as those of normal germination test. The formula used to calculate speed of germination was [10]

(1) $$\begin{array}{c} \text{Mean}\;\text{germination}\;\text{time}\;\left(\text{MGT}\right)=\frac{\sum nd}{\sum n}, \end{array}$$

*n* = number of seeds which germinate on day “*d*”, *d* = number of days counted from the beginning of germination test.

The seedling vigour and vigour index were calculated by plating the seeds on germination paper of dimension 3′′ × 2′′. The seeds were arranged on the paper and kept vertically on the template placed over water in a tray and covered with another tray to prevent entry of light. Observations were taken on the seventh day of sowing. Three replications of 10 seeds each were sown. The length of roots and shoots was measured in centimeter and calculated on the basis of total number of seeds plated [11]. Vigour index was computed adopting method of Abdul-Baki and Anderson [12].

### 2.1. Moisture Determination

The most common method of determination of seed moisture is oven drying method. The principle underlying this method is the elimination of water from the seed by drying precisely for prescribed duration and temperature. The moisture content is expressed as the percentage of the seed dry weight. For determining the seed moisture content, about 1-2 g seed sample was drawn randomly in three replications of each accession. Seeds were dried at 103 ± 2°C for 17 hrs [13]. Each empty moisture bottle was weighed including its lid. Samples were added to these bottles. The weight of the moisture bottle with sample was taken. Samples in moisture bottles with their lids open were placed in oven for drying. At the end of drying period, the bottles were closed with their lids and were allowed to cool for about 30 min. in a dessicator containing silica gel and then weighed again. The moisture contents were calculated using the formula

(2) MM2−M3M2−M1×100=Difference  in  wt.  of  sampleInitial  wt.  of  sample×100,M=moisture  content,M1=weight  of  empty  bottle  with  its  lid,M2=weight  of  bottle  with  lid  and  sample  before  drying,M3=weight  of  bottle  with  lid  and  sample  after  drying.

The data were analyzed statistically by Factorial CRD [14] and difference among means was tested for significance using least significant difference tests, at 1% probability level. The data recorded as percentage were transformed to the respective angular (arc sin) values before subjecting them to statistical scrutiny. The effect of extreme levels of relative humidity (1% and 75%) and intermediate levels (11, 32, and 50%) was analyzed separately in order to critically evaluate the influence of RH on storage behaviour of the accessions under study.

## 3. Results and Discussion

### 3.1. Moisture Content

Moisture content is the primary factor influencing the storability of seeds. Different accessions of *Brassica* had moisture content around 0-1% after 70 days of observation for the seeds kept over acid. The seeds acquired the mean equilibration moisture content after 30 days after treatment and thereafter stabilized in this moisture. But when seeds were subjected to high RH (75%) they acquired 10–12% moisture even within 15 days. *B. napus* and *E. sativa* accessions possessed the highest moisture content compared to all other accessions. When these seeds were subjected to intermediate levels of RH at 11, 32, and 50%, moisture contents were equilibrated around 4-5% in MgCl_2_, more than 5% in CaNO_3_, and less than 3% in LiCl (Table 1).

### 3.2. Germination Percentage

**Figure 1:**
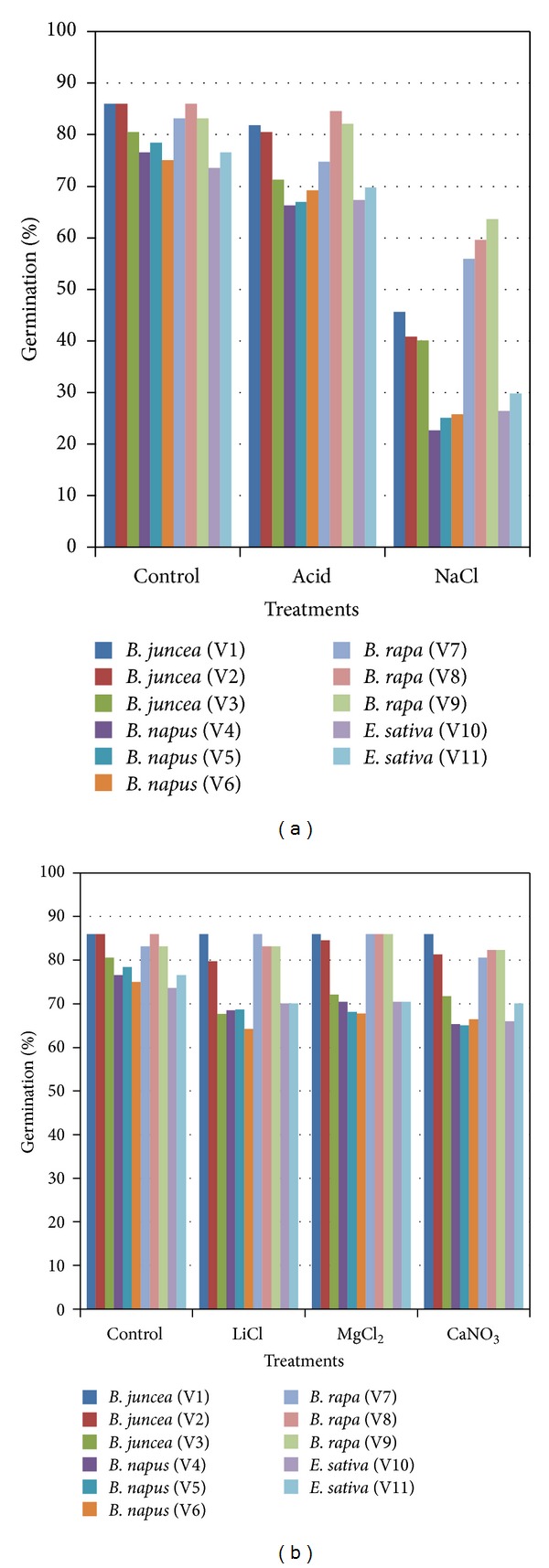
Effect of high and low (a) and intermediate (b) RH on germination percentage of various accessions of *Brassica* spp. and *Eruca sativa*.

When the seed lots of different accessions of *Brassica* spp. and *Eruca sativa* were subjected to storage at elevated temperature (40 ± 2°C) over higher and lower RH (75 and 1%), their survival rates were significantly different (Figures 1(a) and 1(b)). *B. rapa* and *B. juncea* species unusually maintained better viability even after ageing for 90 days, whereas accessions of *B. napus* and *Eruca sativa* had lost most of their viability by this time. The accessions of *B. napus* and *Eruca sativa* had equilibrated to higher moisture content (11-12%) as compared to *B. juncea* and *B. rapa* accessions at high RH. Even small increments in moisture content are expected to influence the storability of seed at elevated temperature significantly and hence the observed differences in the germination percentage of the accessions of various species of *Brassica* and *Eruca sativa*. The variability in seed viability could also be attributed to genotype differences between *Eruca sativa* and *Brassica* spp., as reported by Balesevic-Tubic et al. [15] in soybean and sunflower seeds, as well as several other factors like seed size, 100-seed weight and seed coat colour, and so forth. This clearly indicates that rapeseed storage even for a short period under high relative humidity conditions should be avoided in the tropical region where such high temperature and RH conditions may occur.

In contrast to the results of storage at high RH, the germination percentage of seed stored at very low RH (1%) over concentrated sulphuric acid showed minimal decrease. The equilibrium moisture content of the seeds of various accessions was in the range of 0–0.079. Despite such ultralow moisture contents, seeds did not show drastic differences in their germinability and the rest of accessions maintained germination to the level of 85% or more. Storage studies for longer duration are required to brief out the differences between storability of species under such circumstances. Nutile [16] stored seeds of various kinds of vegetables in extreme dry condition (even to a minimum seed moisture content of 0–4%) and found a decrease in germination percentage of lettuce, onion, cucumber, and cabbage only after four years. The maintenance of germination capacity of various accessions of *Brassica *and *Eruca sativa* may be purely due to their genetic makeup, which also controls the proximate composition of the seeds. Many kinds of seed do not withstand drying to such low levels of moisture [17]. Rapeseeds being oil seeds and hydrophobic in nature probably caused tolerating drying to such low moisture content. Similar work on storage behaviour of *Pongamia pinnata* seeds also revealed comparable results of decline in germination percentage and vigour parameters and also increased time needed for germination [18].

During storage at intermediate levels of relative humidity at 11, 32, and 50% maintained using saturated salt solutions of lithium chloride (LiCl), magnesium chloride (MgCl_2_), and calcium nitrate Ca(NO_3_)_2_, minimum decline in the germination percentages was observed for two accessions of *B. juncea* and all accessions of *B. rapa* and germination percentage above 85% was maintained in accessions of *B. napus* and *E. sativa*. The seed moisture contents to which the seeds equilibrated at these relative humidities were 3, 4, and 5 percent, respectively. Although the differences are less significant, there is an optimum water content for seed storage and for seeds with high oil content. The value of the optimum is less than the bench mark 5% water content, recommended for seed storage by IBPGR [19]. Pradhan and Badola [20], while studying the effect of storage conditions and storage periods of seed germination in *Swertia chirata*, also suggested 4°C as the most appropriate storage temperature for long term storage. There is also undisputed evidence that the value of the critical water content varies among species and has an inverse relationship with the lipid content of seed [2, 6]. *Brassicas*, being rich in oil content, equilibrated to low moisture contents, especially at 11 and 32% RH at 40 ± 2°C.

Nakamura [17] has listed *B. rapa* as one of the species which can tolerate storage below 10%. Halder and Gupta [21] studied the deterioration patterns of sunflower seeds stored at low and high relative humidities. They observed that the germinability changed very little during storage but observed reduced level of dehydrogenase enzymes in the embryonal axis of seeds at both low and high RH which indicates that changes in macromolecules occurred even when no observable changes could be seen at low relative humidity level. On the other hand, Dey and Mukherjee [22] while investigating the effects of different relative humidities (95 and 36%) on the storability of maize and mustard seeds at elevated temperature of 44°C observed detrimental effect of low RH on germination and seedling growth. Accelerated rate of production of lipid peroxidation products and decline in phospholipids have been cited as reason for faster rate of deterioration of seed stored at lower relative humidities at elevated temperature.

### 3.3. Mean Germination Time (MGT)

MGT was significantly different among the species but not among accessions within the species. Storage at high RH (75%) and different accessions of *Brassica *spp. and *Eruca sativa* led to highly significant increase in the MGT (Tables 2(a) and 2(b)). Delayed germination is one of the earliest responses exhibited by crop seeds, when they deteriorate. The increase in MGT was significantly higher for *B. napus* and *Eruca sativa* as compared to *B. juncea* and *B. rapa* accessions. The high MGT was reflected by low germination percentage in high RH treatment. Even though germination percentage was maintained high at all the three intermediate RH levels, time taken for germination (MGT) was high in all the accessions compared to the control.

According to Bailly et al. [23], the cause of increasing mean time to germination probably is due to delay in the process when germination started, since repairing the membrane damage and other parts of cells and also restarting antioxidant system activity and preventing oxidative stress are time consuming processes. It was stated that the rate at which the seed ageing process takes place depends on the ability of the seeds to resist degradation changes and protection mechanisms, which are specific for each plant species [24–26] and also differ among varieties of the same species [27]. Increased rates of deterioration at elevated temperature and higher moisture contents have been reported, for corn seeds aged under similar conditions by Tang et al. [28] and sunflower seeds by Pardhan and Badola [20]. Storage at very low RH (1%) also increased the mean germination time significantly but the values were smaller.

### 3.4. Shoot and Root Length of Seedlings

The decline in shoot length was more remarkable at higher RH treatment. *Brassica napus* had the least shoot growth at 75% RH (Tables 3(a) and 3(b)). The results indicated that storage under very low RH for a period of three months is not as detrimental as that under 75% RH over NaCl. In the table, the shoot length of control seeds ranged from 5.08 to 10.92 cm while under 1% RH the range was 6.11 to 7.6 cm. The differences are significant but not drastic. On the other hand, at high RH (75%) the shoot length was very much reduced, the range between various accessions being 0.6–2.32 which is 5–10-fold as compared to control lots. Minimum shoot length was observed for the *Eruca sativa* accessions followed by *B. napus* accessions. Differences among the different accessions of a species were not significant although difference between accessions of different species was highly significant.

Similar to shoot length root length declined with the passage of time, both under high and low RH (Tables 4(a) and 4(b)). There was sharp decline in mean root length at higher RH treatment as against the low RH treatment, where the decline in shoot length was not drastic. The decline in root vigour was similar to that for shoot vigour but of lower magnitude than those seeds maintained under low RH (1%) which lost little vigour during three months of storage. Hence both shoot and root length decreased reasonably when seeds were stored at higher RH (75%). The trends were similar to those for germination and MGT where *B. napus* and *Eruca* sp. accessions recorded greater decline in root and shoot vigour than *B. juncea* and *B. rapa* accessions. Shoot vigour was more sensitive to storage as revealed by significant decline as compared to the respective controls in all the accessions.

Seeds stored at high moisture content demonstrate increased respiration, heating, and fungal invasion resulting in reduced seed vigour and viability [29]. Comparatively lesser initial vigour could have been responsible for the greater differences observed for the accessions, which recorded greater decline after deterioration. Parallel decline in seedling vigour along with loss of germination has been reported by Shanmughavel et al. [30] for soybean, Joao Abba and Lovato [31] in maize seeds, and Saxena et al. [32] in Sesamum seeds. At 1 *percent* RH, on the other hand, the changes in the vigour parameters were of lesser magnitude. Genetic factors and seed chemical composition influence the expression of seed deterioration and vigour decline [33, 34] and are indicated by the differential response of various accessions of species to different types of ageing. After 90 days of storage of *B. juncea* seeds at 36% RH, Halder and Gupta [21] observed nearly fourfold decrease in root and shoot vigour. But in the present investigation, none of the accessions of *Brassica* spp. or *Eruca sativa* registered such levels of deterioration at relative humidities ranging from 11 to 50%.

### 3.5. Vigour Index

**Figure 2:**
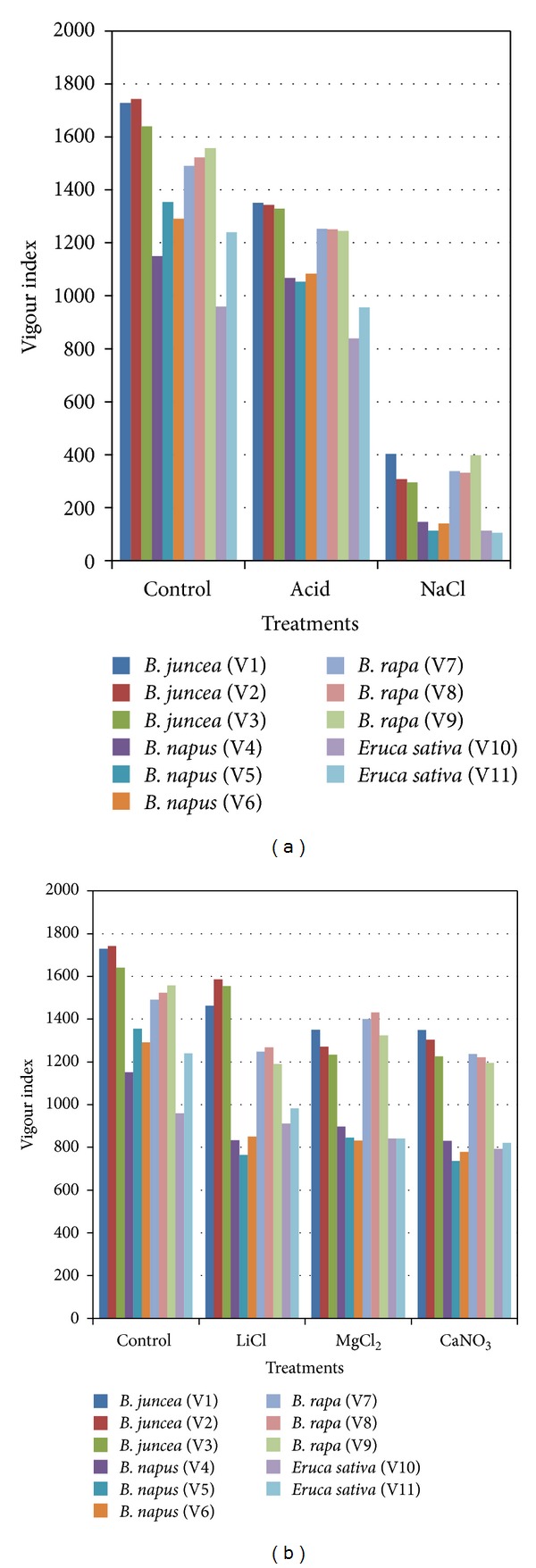
Effect of high and low (a) and intermediate (b) RH on vigour index of various accessions of *Brassica* spp. and *Eruca sativa*.

The changes in seedling vigour index also closely followed the changes in viability, since vigour index is directly depending on germination percentage and seedling vigour. The effect of high RH was very drastic on all varieties irrespective of the species. This trend was in contrast to that observed for seeds stored under low RH. With the progress of ageing, significant reduction in the vigour index was observed. The initial vigour index was maximum in the untreated seeds of all the accessions studied. Storage under 1% RH resulted in small but significant fall in the vigour index of the accessions analyzed. Maximum decline in the parameter was recorded for *Eruca sativa* (105–111.85) (as much as ten times) followed by *B. napus* accessions (112–145.29) (approximately 9 times) in the seed lot stored under 75% RH at 40 ± 2°C. Comparison of overall means for storage under low and high RH reflected the least tolerance of the seeds of accessions of *Eruca sativa* to stressful environmental conditions (Figures 2(a) and 2(b)). At intermediate levels of RH, though the vigour index was reduced, but not to a considerable extent (Figure 2).

## 4. Conclusions

The study illustrated the role of relative humidity in maintaining seed moisture content while processing and storing seeds of *Brassica* spp. and *Eruca sativa*. Germination percentage and mean germination time were drastically affected at high RH than low RH irrespective of the species. The above trend observed was true for vigour index also since vigour index is a consequence of germination percentage and seedling vigour. Hence storage of seeds with high moisture content (10–12%) caused faster deterioration in all accessions and was least at 32% RH, maintaining seed moisture content of 3% without having any adverse effect on physiological parameters. On analyzing the variability in seed storage behaviour, it was observed that germination percentage was recorded maximum in *B. rapa* followed by *B. juncea* and minimum in seeds of *B. napus* and *E. sativa*. Mean germination time was least for *B. rapa* and *B. juncea* at levels of relative humidity. In *B. napus* and *E. sativa* seeds, MGT had increased significantly for seeds subjected to high RH and was least for RH of 32%. Maximum growth of shoot and root was exhibited by *B*. *juncea* followed by *B. rapa* at all levels of relative humidity. Maximum shoot and root length were observed when seeds were stored at a seed moisture content of 3%. Thus, *B. rapa* and *B. juncea* showed more tolerance towards adverse storage conditions than *B. napus* and *Eruca sativa* indicating their physiological stability. Hence, it is convincingly proved that relative humidity plays an important role in maintaining the seed moisture content and thus directly affecting seed viability.

## Figures and Tables

**Table 1:** Equilibration moisture contents of various accessions of *Brassica *spp. and *Eruca sativa*.

	*B. juncea* (V1)	*B. juncea* (V2)	*B. juncea* (V3)	*B. napus* (V4)	*B. napus* (V5)	*B. napus* (V6)	*B. rapa* (V7)	*B. rapa* (V8)	*B. rapa* (V9)	*E. sativa* (V10)	*E. sativa* (V11)
	(%)	(%)	(%)	(%)	(%)	(%)	(%)	(%)	(%)	(%)	(%)
Control	5.8	6	5.5	6.1	5.8	5.6	5.6	5.7	5.7	8.5	8.1
Acid (1%)	0.04	0.06	0.11	0.09	0.02	0.03	0.12	0.05	0.08	0.12	0.08
LiCl (11%)	3.03	2.99	2.88	3.63	3.46	4.36	3.43	3.24	3.01	3.8	3.9
MgCl_2_ (32%)	4.52	4.27	4.21	4.92	5.01	4.82	4.25	4.24	4.56	4.78	5.43
CaNO_3_ (50%)	5.08	5.1	5.06	5.31	5.58	5.84	5.25	5.35	4.91	5.71	5.37
NaCl (75%)	10.91	10.57	11.65	12.85	11.71	11.36	10.9	10.63	11.12	12.54	11.92

**Table:** (a)

	Control (days)	Acid 1% (days)	NaCl 75% (days)	Mean (days)
*B. juncea* (V1)	1.01	1.99	2.62	2.22
*B. juncea* (V2)	1.06	2.03	2.25	2.07
*B. juncea* (V3)	1.2	2.08	1.98	1.97
*B. napus* (V4)	1.05	2.1	0.86	1.45
*B. napus* (V5)	1.04	2.14	0.93	1.5
*B. napus* (V6)	1.08	2.08	0.87	1.45
*B. rapa* (V7)	1.01	2.02	3.52	2.65
*B. rapa* (V8)	1.04	2	3.48	2.61
*B. rapa* (V9)	1.01	2.02	3.34	2.5
*E. sativa* (V10)	1.12	2.11	1.58	1.8
*E. sativa* (V11)	1.14	2.13	1.77	1.89

Mean	1.07	2.06	2.11	
CD (*P* = 0.01)				
Treatment 0.29				
Variety 0.38				

**Table:** (b)

	Control (days)	LiCl 11% (days)	MgCl_2_ 32% (days)	CaNO_3_ 50% (days)	Mean (days)
*B. juncea* (V1)	1.01	2.02	2.06	2.03	1.93
*B. juncea* (V2)	1.06	2.13	2.07	2.09	1.99
*B. juncea* (V3)	1.20	2.48	2.06	2.12	2.12
*B. napus* (V4)	1.05	2.54	2.06	2.15	2.13
*B. napus* (V5)	1.04	2.48	2.11	2.25	2.16
*B. napus* (V6)	1.08	2.47	2.11	2.14	2.13
*B. rapa* (V7)	1.01	2.02	2.02	2.03	1.92
*B. rapa* (V8)	1.04	2.02	2.02	2.01	1.92
*B. rapa* (V9)	1.01	2.02	2.00	2.04	1.92
*E. sativa* (V10)	1.12	2.24	2.10	2.11	2.05
*E. sativa* (V11)	1.14	2.13	2.09	2.13	2.02

Mean	1.07	2.23	2.06	2.10	
CD (*P* = 0.01)					
Treatment 0.02					
Variety 0.03					

**Table:** (a)

	Control (cm)	Acid (1%) (cm)	NaCl (75%) (cm)	Mean (cm)
*B. juncea* (V1)	10.06	7.56	2.1	5.18
*B. juncea* (V2)	10.92	7.29	1.66	4.91
*B. juncea* (V3)	9.9	7.23	1.72	4.84
*B. napus* (V4)	9.57	7.15	1.16	4.51
*B. napus* (V5)	10.42	7.73	0.9	4.72
*B. napus* (V6)	9.94	7.6	0.97	4.67
*B. rapa* (V7)	9.21	7.53	2.07	5.09
*B. rapa* (V8)	8.68	7.35	2.28	5.07
*B. rapa* (V9)	9.58	7.58	2.32	5.26
*E. sativa* (V10)	5.08	5.76	0.6	3.31
*E. sativa* (V11)	7.47	6.11	0.68	3.67

Mean	9.16	7.17	1.5	
CD (*P* = 0.01)				
Treatment 0.22				
Variety 0.28				

**Table:** (b)

	Control (cm)	LiCl (11%) (cm)	MgCl_2_ (32%) (cm)	CaNO_3_ (50%) (cm)	Mean (cm)
*B. juncea* (V1)	10.06	8.16	8.25	9	8.63
*B. juncea* (V2)	10.92	8.2	7.8	8.54	8.46
*B. juncea* (V3)	9.9	8.39	7.9	8.83	8.52
*B. napus* (V4)	9.51	7.3	6.41	6.84	7.12
*B. napus* (V5)	10.42	6.68	7.2	6.24	7.08
*B. napus* (V6)	9.94	7.16	6.84	6.3	7.09
*B. rapa* (V7)	9.21	6.68	7.66	7.04	7.34
*B. rapa* (V8)	8.68	6.06	8.65	6.57	7.25
*B. rapa* (V9)	9.58	6.28	8.48	7.14	7.53
*E. sativa* (V10)	5.08	6.37	7.4	6.03	6.45
*E. sativa* (V11)	7.47	6.83	7.81	5.82	6.88

Mean	9.16	7.1	7.67	7.12	
CD (*P* = 0.01)					
Treatment 0.27					
Variety 0.4					

**Table:** (a)

	Control	Acid 1% (cm)	NaCl 75% (cm)	Mean (cm)
*B. juncea* (V1)	7.45	6.72	2	4.57
*B. juncea* (V2)	8.17	6.28	1.66	4.25
*B. juncea* (V3)	7.11	6.18	1.73	4.17
*B. napus* (V4)	3.26	3.4	0.46	2.02
*B. napus* (V5)	3.45	3.7	0.4	2.15
*B. napus* (V6)	3.58	3.64	0.38	2.12
*B. rapa* (V7)	5.54	5.64	1.79	3.84
*B. rapa* (V8)	5.88	5.22	1.56	3.74
*B. rapa* (V9)	5.52	5.46	1.77	3.56
*E. sativa* (V10)	4.69	3.7	0.66	2.35
*E. sativa* (V11)	5.26	3.52	0.8	2.37

Mean	4.86	4.86	1.2	
CD (*P* = 0.01)				
Treatment 0.24				
Variety 0.31				

**Table:** (b)

	Control	LiCl 11% (cm)	MgCl_2_ 32% (cm)	CaNO_3_50% (cm)	Mean (cm)
*B. juncea* (V1)	7.45	7.96	7.09	7.7	7.51
*B. juncea* (V2)	8.17	7.48	7.82	7.55	7.61
*B. juncea* (V3)	7.11	7.76	7.12	7.78	7.5
*B. napus* (V4)	3.26	3.31	3.34	4.2	3.58
*B. napus* (V5)	3.45	3.06	3.66	3.36	3.37
*B. napus* (V6)	3.58	3.6	3.45	3.43	3.5
*B. rapa* (V7)	5.54	3.97	6.59	4.84	5.18
*B. rapa* (V8)	5.88	4.09	6.67	5.1	5.24
*B. rapa* (V9)	5.52	4.53	6.72	5.2	5.41
*E. sativa* (V10)	4.69	5.31	6.74	5.13	5.62
*E. sativa* (V11)	5.26	6.38	6.41	4.24	5.6

Mean	5.44	5.22	5.96	5.32	
CD (*P* = 0.01)					
Treatment 0.27					
Variety 0.4					
